# A100 A PROVINCIAL APPROACH TO ASSESSING ENDOSCOPY PATIENT EXPERIENCE ENHANCES SITE PARTICIPATION AND CANADA-GLOBAL RATING SCALE COMPLIANCE

**DOI:** 10.1093/jcag/gwab049.099

**Published:** 2022-02-21

**Authors:** C Oilund, L Morrin, B Moysey, S Jelinski, J Snider, F Underwood, C Spankie, N Nemecek, M Greenaway

**Affiliations:** 1 Digestive Health Strategic Clinical Network, Alberta Health Services, Edmonton, AB, Canada; 2 University of Calgary, Calgary, AB, Canada; 3 Alberta Health Services, Edmonton, AB, Canada

## Abstract

**Background:**

The Canada-Global Rating Scale (C-GRS) is a web-based, patient centered endoscopy quality improvement tool. It assesses the quality of services a unit provides in two dimensions: clinical quality and patient experience. Endoscopy units submit results to the Canadian Association of Gastroenterology twice a year. In Alberta, units receive an A, B, C or D grade for each of the 12 C-GRS Items and a C-GRS score.

The C-GRS promotes that patient feedback is sought annually. Patient feedback is important because it informs practice improvement opportunities. However, survey creation, distribution, analysis and reporting can be time consuming and costly for an endoscopy unit and is a potential barrier to participation.

**Aims:**

The purpose of this quality improvement project is to demonstrate how a provincial infrastructure, which includes coordination, management and reporting of an endoscopy patient satisfaction survey, can enhance provincial endoscopy unit survey participation and facilitate C-GRS compliance.

**Methods:**

The Digestive Health Strategic Clinical Network (DHSCN), the Alberta Colorectal Cancer Screening Program and Primary Data Support (PDS) collaborated on the Provincial Endoscopy Patient Experience Survey (PEPES) in 2019. An existing paper survey was adapted to meet the needs of the 50 endoscopy units in AB with the addition of an electronic version. Education about the PEPES process was provided via a webinar and site visits. Each unit was responsible for distribution of the surveys to their patients. PDS coordinated the paper survey process and the DHSCN managed the electronic survey submissions. Paper survey results were merged with electronic PEPES data. A summary report was provided to units and shared with each AHS Zone Endoscopy Executive Leadership Team.

**Results:**

Provincially coordinated implementation of the PEPES fosters compliance with C-GRS criteria. Participating endoscopy units were able to achieve at minimum 9 C-GRS descriptors and improve their C-GRS score in the following 7 of the 12 C-GRS Items: consent, comfort, equality, booking, privacy, aftercare and feedback.

Initial enrollment in the PEPES increased with the onset of provincial coordination (Figure 1). However, subsequent participation was negatively impacted by COVID-19 as many endoscopy units in AB were required to decrease their capacity and redeploy staff.

**Conclusions:**

A provincially coordinated approach to the management of an endoscopy patient experience survey is an effective way to enhance site participation and improve C-GRS scores. Units can focus on actioning survey results, rather than the burden of survey administration. Future work includes comparison of results across sites allowing for potential provincial equity issues to be addressed.

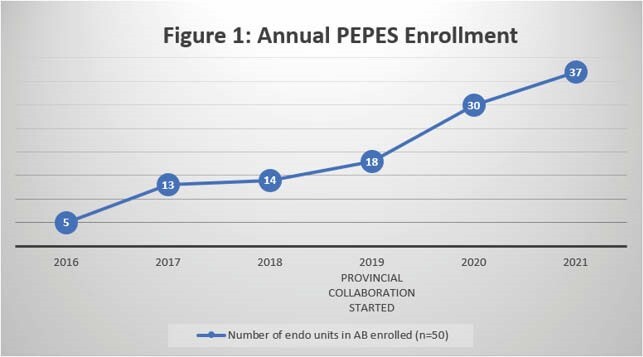

**Funding Agencies:**

None

